# Effects of Cariprazine, Aripiprazole, and Olanzapine on Mouse Fibroblast Culture: Changes in Adiponectin Contents in Supernatants, Triglyceride Accumulation, and Peroxisome Proliferator-Activated Receptor-γ Expression

**DOI:** 10.3390/medicina55050160

**Published:** 2019-05-17

**Authors:** László-István Bába, Melinda Kolcsár, Imre Zoltán Kun, Zsófia Ulakcsai, Fruzsina Bagaméry, Éva Szökő, Tamás Tábi, Zsolt Gáll

**Affiliations:** 1 Department of Pharmacology and Clinical Pharmacy, Faculty of Pharmacy, University of Medicine, Pharmacy, Sciences and Technology of Tîrgu Mureș, 540139 Tîrgu Mureș, Romania; laszlo.baba@umfst.ro (L.-I.B.); zsolt.gall@umfst.ro (Z.G.); 2 Doctoral School, Faculty of Medicine, University of Medicine, Pharmacy, Sciences and Technology of Tîrgu Mureș, 540139 Tîrgu Mureș, Romania; kunimre@gmail.com; 3 Department of Pharmacodynamics, Semmelweis University, 1089 Budapest, Hungary; ulakcsaizs@gmail.com (Z.U.); bagamery.fruzsina@pharma.semmelweis-univ.hu (F.B.); szoko.eva@pharma.semmelweis-univ.hu (É.S.); tabi.tamas@pharma.semmelweis-univ.hu (T.T.)

**Keywords:** adipogenesis, triglyceride accumulation, peroxisome proliferator-activated receptor-γ expression, adiponectin, antipsychotics

## Abstract

*Background and Objectives*: The use of the dopamine-partial agonist subclass (also termed dopamine stabilizers) of atypical antipsychotics for the treatment of negative schizophrenia symptoms and some mood disorders has increased recently. Similar to other second-generation antipsychotics (SGAs), aripiprazole (ARI) and cariprazine (CAR) also influence food intake, but the peripheral effects of these drugs on adipose–tissue homeostasis, including adipokine secretion as well as lipo- and adipogenesis, are not fully elucidated. In this study, we explored the adipocyte-related mechanisms induced by second-generation antipsychotics (SGAs), leading to changes in peripheral signals involved in energy homeostasis. *Materials and Methods:* CAR, a new SGA, was compared with ARI and olanzapine (OLA), using cell cultures to study adipogenesis, and the expression levels of peroxisome proliferator-activated receptor-γ (PPAR-γ) was measured in adipocytes derived from mouse fibroblasts, by western blotting on days 7, 14, and 21 postinduction. The triglyceride (TG) content of the cells was also evaluated on day 15 using Oil Red O staining, and the adiponectin (AN) content in the cell culture supernatants was quantified on days 7 and 15 by enzyme-linked immunosorbent assay. Cells were treated with two concentrations of ARI (0.5 and 20 µg/mL), OLA (1 and 20 µg/mL), and CAR (0.1 and 2 µg/mL). *Results:* Both concentrations of ARI and OLA, as well as the lower concentration of CAR, significantly increased the TG contents. The AN levels in the supernatants were significantly increased by the higher concentration of ARI on days 7 and 15 (*p* < 0.05). Although PPAR-γ levels were not significantly affected by ARI and OLA, the lower concentration of CAR induced a significant time-dependent decrease in PPAR-γ expression (*p* < 0.05). *Conclusions:* The in vitro adipogenesis considered from TG accumulation, AN secretion, and PPAR-γ expression was differently influenced by ARI, CAR, and OLA. Understanding the adipocyte-related mechanisms of antipsychotics could contribute to understanding their weight-influencing effect.

## 1. Introduction

Second-generation antipsychotics (SGAs) include newer agents used in the treatment of several psychiatric diseases, and are considered to be different from older drugs (also called classical or typical antipsychotics). These agents are also thought to be more favorable in terms of efficacy and side effects; for example, SGAs do not induce the characteristic extrapyramidal side effects (EPS) and are more effective in treating the negative signs of schizophrenia [1,2,3,4]. Although SGAs do not induce the most commonly reported adverse effects of first-generation antipsychotics (e.g., EPS, sedation, cardiovascular effects, anticholinergic effects, and hyperprolactinemia), they are characterized by metabolic effects, such as weight gain, increased blood glucose, and increased plasma lipid levels [4]. Weight gain is not thought to be dose-dependent within the normal therapeutic dose range, whereas the glycemic abnormalities may comprise mild (e.g., worsening of insulin resistance in patients with already compromised glycemic control) to very severe forms of ketoacidosis or hyperosmolar coma [4,5].

Based on extensive evidence published to date, different SGAs show varying inductions of adverse metabolic events [5,6,7,8,9,10]. The proposed mechanisms of antipsychotic-induced weight gain involve multiple pathways, including antagonism of 5HT_2C_, H_1_, and D_2_ receptors associated with increased insulin resistance, as well as reduced glucose uptake by skeletal muscles [11]. The most prominent effects are produced by olanzapine (OLA) and clozapine, whereas much lower or even unnoticeable effects are observed following treatment with amisulpride or aripiprazole (ARI), which can be considered “metabolically neutral” under certain circumstances [6,7,9,12]. Notably, the molecular pharmacology of SGAs varies greatly in terms of binding profiles and having very different effects on various serotonergic (5-HT), dopaminergic, histaminergic (H), adrenergic, and cholinergic receptors [13]. Some authors consider the H_1_ receptor affinity as the most important predictor of short-term weight gain [14]. Correspondingly, the most orexigenic SGA (i.e., OLA) is a 5HT_2C_ and H_1_ antagonist with high affinity (Ki = 6.8 and 2 nM for 5HT_2C_ and H_1_, respectively) [14,15]. ARI and cariprazine (CAR) have considerably lower affinity towards these receptors (Ki = 15 and 61 nM for 5HT_2C_ and H_1_, respectively, for ARI; Ki = 134 and 23.2 nM for 5HT_2C_ and H_1_, respectively, for CAR) [16]. Moreover, ARI and CAR are both partial agonists at D_2_ and D_3_—the main difference between them being that CAR has nearly 10-fold higher affinity towards D_3_ than D_2_ [16]. This can partially explain the metabolic differences between CAR and ARI.

Adipose tissue is no longer considered an inert organ, and is now known to have a role in storing energy in the form of triglycerides (TGs) [17]. Moreover, the adipose tissue secretes bioactive signaling molecules, mainly proteins, being a part of the endocrine signaling system. These molecules are commonly termed “adipokines”, indicating factors that are secreted by adipocytes and delivered to the systemic circulation. Adiponectin (AN) is an important adipokine whose concentration is inversely related to adiposity and insulin resistance [18]. Indeed, hypoadiponectinemia is associated with dyslipidemia, hypertension, oxidative stress, and a carbohydrate-rich diet [19,20]. Furthermore, administration of AN improves insulin sensitivity both under in vitro and in vivo conditions through various mechanisms, e.g., increasing glucose utilization in muscle, inducing fatty acid oxidation in hepatic cells, and decreasing hepatic glucose production; it also decreases body weight in vivo [21,22,23]. These effects are at least partially mediated by a peroxisome proliferator-activated receptor (PPAR)-α, which is involved in the regulation of many genes in different metabolic pathways (e.g., fatty acid oxidation, mitochondrial biogenesis, glucose homeostasis, and inflammation) [18,24].

PPARs are members of the nuclear hormone receptor superfamily, comprising ligand-activated transcription factors that bind to the promoter regions of certain genes and then increase or decrease DNA transcription [25]. PPAR-γ is the main factor involved in adipogenesis and is essential for differentiation; indeed, lack of PPAR-γ expression blocks the differentiation of pre-adipocytes into mature adipocytes [25]. During adipocyte differentiation, the expression of PPAR-γ is markedly increased, thereby promoting differentiation [26]. A number of endogenous ligands bind to PPAR-γ, including fatty acids, prostaglandins, and oxidized phospholipids, and several exogenous compounds, including thiazolidindiones, which enhance insulin sensitivity, are used in the treatment of diabetes mellitus. Some SGAs (OLA, clozapine, risperidone) induce the expression of sterol regulatory element-binding protein (SREBP), leading to enhanced lipogenesis and adipogenesis [27,28,29]. Notably, crosstalk between SREBP and PPAR-γ activation has also been demonstrated [30]. Furthermore, OLA directly stimulates the expression and activation of PPAR-γ (although to a lesser extent than that of SREBP-1) [27]. Interestingly, the expression levels of genes encoding PPAR-γ and PPAR-α are two- to three-fold higher in human adipose tissue-derived stem cells under in vitro conditions after SGA treatment [31]. To the best of our knowledge, no studies have reported the expression of these key adipogenesis factors after CAR treatment or compared the three SGAs.

As recently hypothesized, adipocytes are potential sources of metabolic abnormalities, as observed in SGA-induced metabolic syndrome (MS) [12]. Therefore, in order to assess the contributions of adipocytes to MS, we measured the expression of PPAR-γ, the TG contents of adipocytes, and the AN concentrations of cell culture supernatants during in vitro adipogenesis. Additionally, to evaluate the involvement of CAR in metabolic regulation at the adipocyte level, the results were compared with those of OLA and ARI—i.e., SGAs with the least and most favorable metabolic profiles, respectively.

## 2. Materials and Methods

### 2.1. Cell Culture

The effects of the three SGAs on adipogenesis were studied using mouse embryonic fibroblasts (MEFs). The cell culture was established according to previously published protocol [32]. Cells were cultured to confluency in Dulbecco’s modified Eagle’s medium (DMEM) containing 4 g/L glucose, 10% fetal bovine serum (FBS), and 8 µg/dL gentamicin. Differentiation of two-day post-confluent fibroblast cultures was then initiated by addition the induction solution (IND) (0.2 mM isobutylmethylxanthine (IBMX), 0.5 µM dexamethasone, 10 µg/dL insulin, and 10 µM pioglitazone), for two days. Following the two-day induction, the cell lines were refed every two days with the maintenance medium (DMEM + 10% FBS + 10 µg/dL insulin) plus the SGAs at two concentrations (ARI: 0.5 and 20 µM; CAR: 0.1 and 2 µM; OLA: 1 and 20 µM). The lower concentrations of drugs were chosen to be similar or slightly greater than the plasma concentrations seen in normal clinical settings. In this case, the plasma concentration for ARI was 0.47 ± 0.31 µM (for doses of 20 ± 8 mg/day), for OLA it was 0.11 ± 0.08 µM (for doses of 14.2 ± 5.4 mg/day), and for CAR it was about 0.1 µM (for a typical dose of 9 mg/day) [33,34,35]. The higher concentrations of the drugs were chosen in order to maximize the effect of the drugs on the process of adipogenesis, without affecting the viability of the cells. The experiment lasted for 15 days for AN and TG measurement, or 21 days for PPAR-γ assays. As a negative control, only IND was added.

### 2.2. Triglyceride Content Determination of Adipocytes

Assessment of the TG content of the adipocytes was performed on day 15 postinduction and was used as an indicator of the lipid contents of the cells at the middle stage of adipogenesis. The technique chosen for TG measurement was staining with Oil Red O, followed by spectrophotometry. Briefly, the cells were fixed in 8% paraformaldehyde solution for 1 h, washed with distilled water and 60% isopropanol two times, and dried. Cells then were stained with 0.35% Oil Red O solution in isopropanol. After removing the excess stain by washing four times with distilled water, the retained stain was extracted to isopropanol, and its concentration was determined by measuring its absorbance at 500 nm. Because the absorbance of the extract is proportional to the concentration in the Oil Red O stain (which was bound to the lipids within the cells), the absorbance had a direct relationship with the amount of TG accumulated in the cells [36].

### 2.3. Adiponectin Concentration of Cell Culture Supernatant

The AN concentration was determined using a specific enzyme-linked immunosorbent assay (ELISA) kit on days 7 and 15, using the cell culture supernatants (calibration range: 0–12 ng/mL). The Novex ELISA kit (KMP0041) was obtained from Thermo-Fisher Scientific (Waltham, MA, United States) and was used according to the recommendations of the manufacturer.

### 2.4. Peroxisome Proliferator-Activated Receptor-γ Expression

The expression of PPAR-γ was assayed by western blotting on days 7, 14, and 21. Glyceraldehyde-3-phosphate dehydrogenase (GAPDH), which is ubiquitously expressed in most cell types and is therefore a robust standard for western blot measurements, was used as a loading control. Total cell lysates were prepared using a radio-immunoprecipitation assay buffer containing 50 mM Tris-HCl (pH 8.0), 150 mM NaCl, 1% Nonidet P-40, 0.5% sodium deoxycholate, and 0.1% sodium dodecyl sulfate (SDS). After denaturation by heating at 95 °C for 5 min in Laemmli buffer (0.1% 2-mercaptoethanol, 0.0005% bromophenol blue, 10% glycerol, 2% SDS, and 63 mM Tris-HCl (pH 6.8)), 30 µg of protein samples were separated in 15% SDS-polyacrylamide gels and then transferred onto polyvinylidene difluoride (PVDF) membranes. Membranes were then blocked with 5% non-fat dry milk dissolved in Tris-buffered saline containing 0.1% Tween 20 (TBST) for 1 h, and were then probed overnight with primary antibodies for PPAR-γ (1:500 dilution, sc-7196; Santa-Cruz Biotechnology, Dallas, TX, United States) or GAPDH (1:10,000 dilution, MAB5718; R&D Systems, Minneapolis, MN, United States) in the same solution at 4 °C. After washing with TBST three times, membranes were incubated with 1:2000 diluted HRP-conjugated secondary antibody for 1 h at room temperature. The proteins were detected on autoradiography films using enhanced chemiluminescence (ECL) reagent. The films were scanned, and the obtained images were evaluated using ImageJ software (v. 1.51j8, National Institute of Health, Bethesda, MD, United States), and the amount of protein was proportional to the size of the black spots. The quantity of PPAR-γ was expressed as the ratio of PPAR-γ to GAPDH.

### 2.5. Statistical Analysis

The results were analyzed with GraphPad Prism 5, and the level of statistical significance was set at α = 0.05 for all tests. Two-way ANOVA with Dunnett’s post-hoc test was used to test the influence of treatment and dose on the TG content of adipocytes. A three-way ANOVA with Dunnett’s multiple comparisons test was performed to assess the influence of treatment, dose, and time on AN content of the supernatant. For PPAR-γ expression, a two-way ANOVA could not have been performed because of the low number of measurements (*n* = 3), and non-Gaussian distribution of the data. In order to assess the effect of the time and dosage, a series of non-parametric one-way ANOVA, i.e., Kruskall–Wallis (KW) tests, were performed.

## 3. Results

### 3.1. Triglyceride Content of Cells

**Figure 1:**
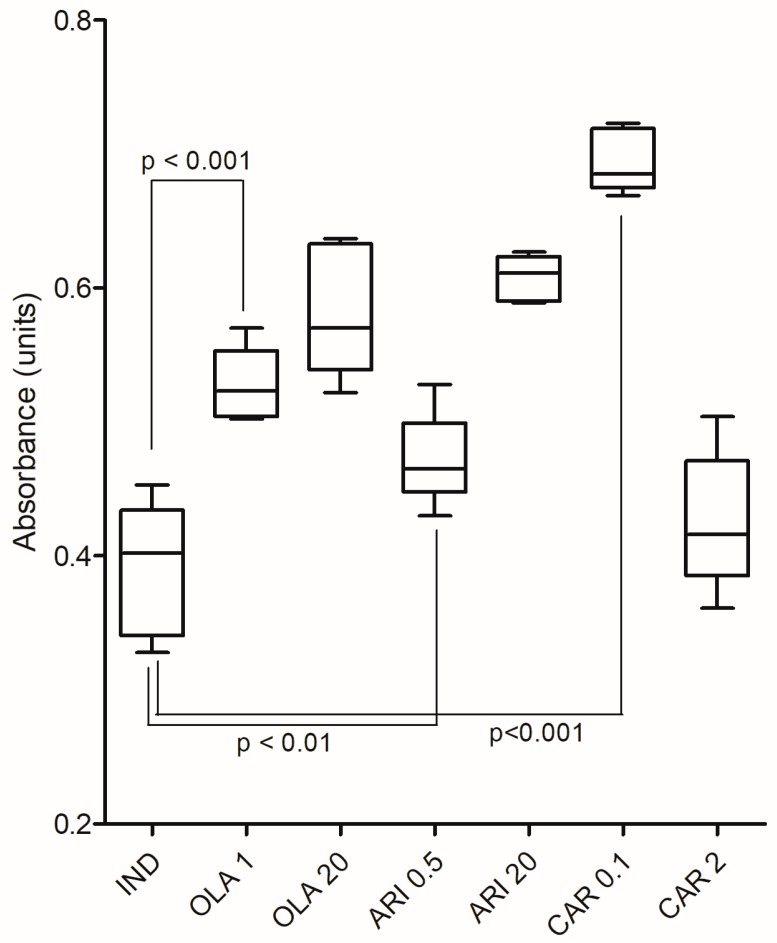
The triglyceride (TG) content of adipocytes after olanzapine (OLA), aripiprazole (ARI), and cariprazine (CAR) exposure. IND: cell lines induced with Dulbecco’s modified Eagle’s medium (DMEM), isobutylmethylxanthine (IBMX), insulin (INS), and pioglitazone(PIO). OLA 1 and OLA 20: cell lines exposed to 1 or 20 µM olanzapine, respectively. ARI 0.5 and ARI 20: cell lines exposed to 0.5 or 20 µM aripiprazole, respectively. CAR 0.1 and 2: cell lines exposed to 0.1 or 2 µM cariprazine, respectively. Data are expressed as the median with minimum and maximum values; level of significance was set to α = 0.05.

The results of TG content measurement are summarized in Figure 1.

The two-way ANOVA analysis showed a significant influence caused by the treatment (F (3, 32) = 40.98; *p* < 0.0001) and the interaction (F (3, 32) = 47.70; *p* < 0.0001), but not the dose applied (F (1, 32) = 2.293; *p* = 0.1398). The Dunnett’s multiple comparisons test showed that the TG content of adipocytes was increased significantly by both concentrations of OLA (*p* < 0.001 for both) and ARI (*p* < 0.01 and *p* < 0.001 for the lower and higher concentrations, respectively), as well as by the lower concentration of CAR (*p* < 0.001), compared with the IND group. The 0.1 µM concentration of CAR robustly increased TG accumulation, giving the highest values, whereas the higher concentration of the drug produced a value similar to that of the IND group (*p* > 0.05), the lowest of all treated groups.

### 3.2. Adiponectin Contents in Cell Culture Supernatants

**Figure 2:**
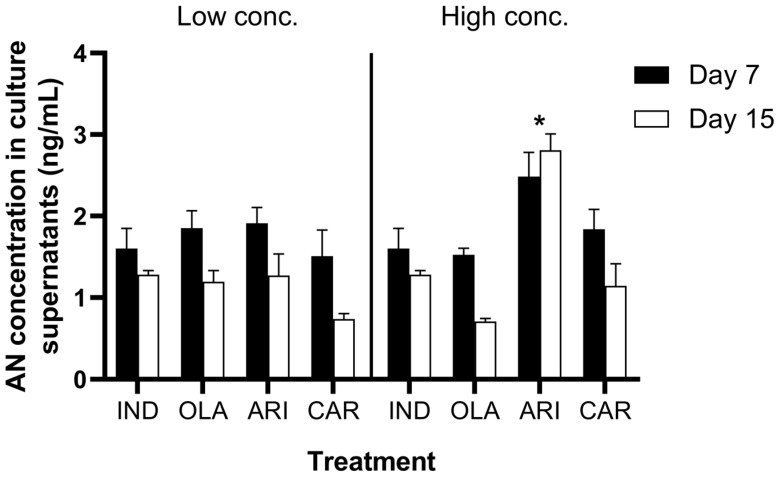
AN concentrations in the cell culture supernatants after 7 and 15 days of treatment. *Significant changes compared with IND (*p* < 0.05). IND: cell lines induced with DMEM, IBMX, insulin (INS), and pioglitazone (PIO). OLA: cell lines exposed to 1 or 20 µM olanzapine. ARI: cell lines exposed to 0.5 or 20 µM aripiprazole. CAR: cell lines exposed to 0.1, 2 µM cariprazine. Data are expressed as means ± standard error of mean, and the level of significance was set to α = 0.05.

A three-way ANOVA showed significant main effects of treatment (F (3, 62) = 13.77, *p* < 0.001), concentration (F (1, 62) = 5.799, *p* = 0.019), and time (F (1, 62) = 21.29, *p* < 0.001) on AN contents in the cell culture supernatants. Subsequent two-way ANOVAs (treatment × time) showed that the effect of treatments was not significant when drugs were applied in low concentrations (F (3, 31) = 1.953, *p* > 0.05). However, a Dunnett’s post-hoc test showed that AN content was significantly increased by the higher concentration of ARI on both days 7 and 15 compared with the IND group (F (3, 31) = 20.96, *p* < 0.001; see Figure 2). Interestingly, when testing for the effect of time (two-way ANOVA treatment × dose), there was no influence of the treatment (F (3, 32) = 2.636, *p* > 0.05) on day 7, but a significant modification was observed on day 15 in the AN contents in the supernatants following treatment (F (3, 30) = 18.94, *p* < 0.001; see Figure 2).

### 3.3. Peroxisome Proliferator-Activated Receptor-γ Expression

**Figure 3:**
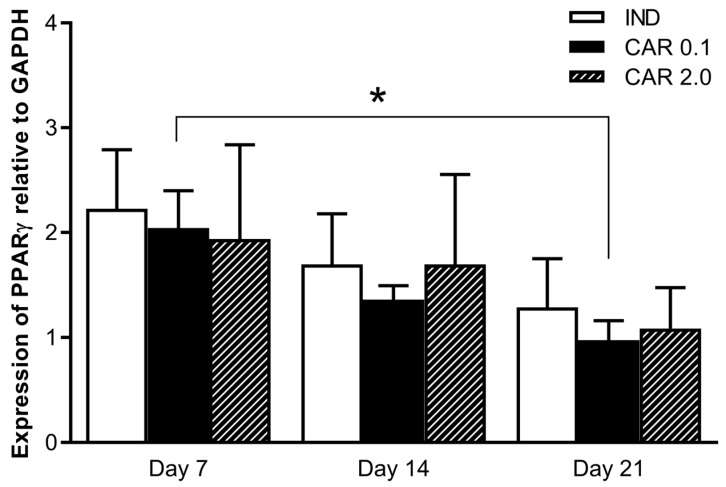
Expression of PPAR-γ in cell cultures after CAR exposure. IND (7, 14, 21): cell lines receiving induction solution (DMEM, IBMX, insulin (INS), and pioglitazone (PIO) for 7, 14, and 21 days, respectively. CAR 0.1, 2 (7, 14, 21): cell lines exposed to 0.1 or 2 µM cariprazine after 7, 14, and 21 days, respectively. Significant changes are noted with * (*p* < 0.05). Data are expressed as means ± standard error of mean, and the level of significance was set to α = 0.05.

PPAR-γ protein expression was normalized to GAPDH protein levels. There were no differences in OLA and ARI groups on any of the testing days. For the CAR groups, a series of non-parametric one-way ANOVA tests were performed, in order to determinate the variation caused by time and dose. Time caused a significant effect in the case of the CAR 0.1 M group (KW value = 5.815, *p* < 0.05). In order to identify the exact source of the difference in time, the Dunn’s multiple comparisons test was used to compare the mean ranks of CAR groups at days 7 and 21, giving a significant difference between day 7 and 21 (2.04 ± 0.79 vs. 0.98 ± 0.37, *p* < 0.05) (Figure 3).

## 4. Discussion

In this study, we examined the effects of SGAs on metabolic regulation at the adipocyte level. Our results showed that these compounds had important effects on TG contents and PPAR-γ expression in adipocytes.

Notably, we found an increase in the TG contents of adipocytes following SGA exposure, consistent with previous findings from rat cell lines [12,37]. Interestingly, the 0.1 µM concentration of CAR robustly increased TG accumulation, whereas the higher concentration did not affect TG levels. Although these data suggest that the lower concentration promoted differentiation, this interpretation must be made carefully, considering the differences between in vitro and in vivo conditions. Although CAR displayed unfavorable effects in this in vitro study, in the clinical setting, CAR induces only modest or even no weight gain at all, even after chronic treatment [16,35,38]. Our results showed that ARI and OLA also stimulated adipogenesis. Importantly, exposure to 0.5 µM ARI slightly increased TG accumulation, displaying a modest but significant effect, whereas the higher concentration of ARI induced a marked increase in TG contents versus that in the IND group. In one previous study, ARI was shown to increase the expression of key adipogenesis-related and pro-inflammatory genes [31]. Moreover, ARI markedly decreased TG accumulation in perirenal adipocytes, decreased lipid vacuole size at lower doses (4 mg/kg), and increased vacuole size at higher doses (8 mg/kg) in rats [39]. The first observation (i.e., the effects of 4 mg/kg ARI) conflicted with clinical reports, whereas the latter was consistent with clinical results, supporting that ARI is an SGA with a favorable metabolic profile [6,7,9,11]. OLA, which has been extensively studied, displays a similar increase in TG content, consistent with previous in vitro findings, suggesting that the adipocyte-related effects of OLA are important and may contribute to OLA-induced MS [27,31,37,40].

CAR significantly decreased PPAR-γ expression in a time-dependent manner. OLA and ARI did not influence PPAR-γ expression. Sárvári et al. found that the mRNA levels of PPAR-γ and other genes were increased after ARI treatment [31]. However, to the best of our knowledge, no studies have described the amount of PPAR-γ protein expressed in mouse pre-adipocytes. Notably, increase in the mRNA and protein levels of a gene are not equal, and the translational process is controlled by several other factors to regulate the end product. Thus, further studies are needed to assess the mechanisms mediating PPAR-γ expression in this context.

Our results showed that within seven days, the AN content in the supernatant was not influenced significantly by the treatment. This observation was consistent with previous reports indicating traceable levels of AN at seven days after induction under in vitro conditions [41]. However, the AN content in the supernatant increased after exposure to 20 µM ARI on day 15. Interestingly, in all the other groups, the AN level in cell culture supernatants decreased significantly on day 15 compared with that on day 7. This was surprising considering that earlier studies demonstrating a steady increase in AN secretion as adipogenesis proceeds in human mesenchymal stromal cells [41]. The results regarding AN content in the supernatant highlighted the potential of ARI to induce AN secretion, an adipokine having beneficial effects, by counteracting insulin resistance and hepatic steatosis through the activation of glucose utilization in different tissues via adenosine monophosphate (AMP)-activated protein kinase and PPAR-α activation [18]. Moreover, AN plays an important role in adipose tissue remodeling into an efficient “metabolic sink”, also improving the sensitivity of adipose tissue to adrenergic stimuli via the β_3_ receptor and thus preventing MS [42]. Specifically, AN favored the increase of subcutaneous TG depots and the decrease of visceral TG [42]. OLA induced an increase in TGs, with a parallel reduction in supernatant AN content. Inversely, CAR induced TG accumulation markedly when applied at a low concentration (but not at a high concentration), and did not affect supernatant AN content. Thus, ARI was found to be an SGA with interesting features—i.e., increasing supernatant AN content and enhancing TG accumulation.

Although the cell line used in this study may help to elucidate some aspects of adipogenesis as part of the clinical profile of SGAs, there are some limitations to these conclusions. First, ARI and CAR have active metabolites presenting high lipophilicity that may contribute to the observed therapeutic effects (dihydro-aripiprazole, as well as desmethyl- and didesmethyl-cariprazine, respectively) [35,43]. Second, the AN content in cell culture supernatants do not necessarily reflect secretion of AN from cells, since the concentration is a net result of the dynamic equilibrium between synthesis and degradation [44]. Third, MEFs are not the standard cell line for studying adipogenesis. The most extensively used is the 3T3-L1 cell line; however, several others, including 3T3-F442A, OP9, and C3H10T1/2 cells have been described in the literature [45]. Despite these limitations, our data clearly supports that ARI induces unique time- and dose-dependent increases in AN supernatant concentration. This beneficial effect of the drug may explain the favorable metabolic profile seen in clinical practice [9,15,16].

## 5. Conclusions

Some CNS-acting drugs lack an apparent binding site at the adipocyte level, but nevertheless may alter adipocyte homeostasis. Although ARI and CAR share some pharmacological targets and mechanisms involved in the antipsychotic action, they affected the in vitro induced adipogenesis in mouse fibroblast cells differently: ARI induced TG accumulation, increased AN concentration in culture supernatants, and did not change PPAR-γ expression; while CAR induced TG accumulation, did not change AN concentrations, and decreased PPAR-γ expression. However, both drugs showed some advantages when compared to OLA, which induced TG accumulation, decreased AN concentration, and did not alter PPAR-γ expression. It can be concluded that a favorable metabolic profile of an SGA may involve several underlying processes. Some of these are beneficial, others are not. Nonetheless, the identification of an adipocyte-specific mechanism could contribute to the understanding of drug-induced weight gain.
